# Phenoplant: a web resource for the exploration of large chlorophyll fluorescence image datasets

**DOI:** 10.1186/s13007-015-0068-4

**Published:** 2015-04-03

**Authors:** Céline Rousseau, Gilles Hunault, Sylvain Gaillard, Julie Bourbeillon, Gregory Montiel, Philippe Simier, Claire Campion, Marie-Agnès Jacques, Etienne Belin, Tristan Boureau

**Affiliations:** PHENOTIC, SFR 4207 QUASAV, F-49045 Angers, France; Université d’Angers, Laboratoire d’Hémodynamique, Interaction Fibrose et Invasivité tumorale hépatique, UPRES 3859, IFR 132, F-49045 Angers, France; Institut de Recherche en Horticulture et Semences, UMR1345, INRA, SFR 4207 QUASAV, F-49071 Beaucouzé, France; Institut de Recherche en Horticulture et Semences, UMR1345, AgroCampus-Ouest, SFR 4207 QUASAV, F-49045 Angers, France; Université de Nantes, Laboratoire de Biologie et de Pathologie Végétales EA 1157, SFR 4207 QUASAV, F-44322 Nantes, France; Institut de Recherche en Horticulture et Semences, UMR1345, Université d’Angers, SFR 4207 QUASAV, F-49045 Angers, France; Laboratoire Angevin de Recherche en Ingénierie des Systèmes (LARIS), Université d’Angers, F-49000 Angers, France

## Abstract

**Background:**

Image analysis is increasingly used in plant phenotyping. Among the various imaging techniques that can be used in plant phenotyping, chlorophyll fluorescence imaging allows imaging of the impact of biotic or abiotic stresses on leaves. Numerous chlorophyll fluorescence parameters may be measured or calculated, but only a few can produce a contrast in a given condition. Therefore, automated procedures that help screening chlorophyll fluorescence image datasets are needed, especially in the perspective of high-throughput plant phenotyping.

**Results:**

We developed an automatic procedure aiming at facilitating the identification of chlorophyll fluorescence parameters impacted on leaves by a stress. First, for each chlorophyll fluorescence parameter, the procedure provides an overview of the data by automatically creating contact sheets of images and/or histograms. Such contact sheets enable a fast comparison of the impact on leaves of various treatments, or of the contrast dynamics during the experiments. Second, based on the global intensity of each chlorophyll fluorescence parameter, the procedure automatically produces radial plots and box plots allowing the user to identify chlorophyll fluorescence parameters that discriminate between treatments. Moreover, basic statistical analysis is automatically generated. Third, for each chlorophyll fluorescence parameter the procedure automatically performs a clustering analysis based on the histograms. This analysis clusters images of plants according to their health status. We applied this procedure to monitor the impact of the inoculation of the root parasitic plant *Phelipanche ramosa* on *Arabidopsis thaliana* ecotypes Col-0 and Ler.

**Conclusions:**

Using this automatic procedure, we identified eight chlorophyll fluorescence parameters discriminating between the two ecotypes of *A. thaliana*, and five impacted by the infection of *Arabidopsis thaliana* by *P. ramosa*. More generally, this procedure may help to identify chlorophyll fluorescence parameters impacted by various types of stresses. We implemented this procedure at http://www.phenoplant.org freely accessible to users of the plant phenotyping community.

**Electronic supplementary material:**

The online version of this article (doi:10.1186/s13007-015-0068-4) contains supplementary material, which is available to authorized users.

## Background

In plant science, computer vision applied to the monitoring of plants receives increasing interest. Measurements based on automatic image analysis provide a calibrated analysis, thereby eliminating any subjectivity of the raters and ensuring reproducibility [1-4]. Moreover, image analysis is more accurate than manual annotations [5,6]. Actually, an image is an association of pixels that display various intensities and create the colors. To each grayscale image corresponds a unique histogram featuring the number of pixels for each level of gray. The histograms allow a pixel-by-pixel analysis. Image analysis is used for characterizing the architecture of plants as well as roots or the venation of leaves [7-9], their tolerance to heavy metals [10], the cold tolerance [11] or the impact of a pathogen [5,6].

Moreover, the automation of image analysis and eventually of image acquisition allows high-throughput phenotyping [1]. Several software or applications devoted to image analysis have been developed to answer specific questions such as the measuring of the area of leaves, plants and grains [12-14], the impact of pests and abiotic stresses [5,12,13] and the callose deposition [15]. In practice, such methods have to be user-friendly and automated to match the needs of the community of biologists, especially when large datasets are treated.

In plant science, imaging can be achieved both by visible imaging (e.g. photographs or scans) and by other types of imaging such as chlorophyll fluorescence, thermographic or hyperspectral imaging. Chlorophyll fluorescence (CF) imaging has been used to study the impact of both biotic and abiotic stresses on photosynthesis and hence on plant physiology [5,11,16-25]. Moreover, CF imaging was reported to allow monitoring phenotypes that are not visible to the human eyes [5,11,22,26,27]. Indeed, CF transients and CF parameters may be measured on plants in dark- and light-adapted states [28-30]. Some CF parameters display a robust contrast between healthy and unhealthy tissues while others seem not to be impacted by stresses [18]. For example, the maximum quantum yield of PSII photochemistry (F_v_/F_m_), measured from dark-adapted leaves, can be used to quantify the severity of symptoms induced on bean by *Xanthomonas fuscans* subsp. *fuscans* [5] or to monitor the *Arabidopsis* health status in various drought stress conditions [31]. Some CF parameters from light-adapted leaves, such as the effective quantum efficiency of PSII (Φ_PSII_) and the non-photochemical quenching (NPQ), may also be good discriminating parameters between drought-resistant and drought-sensitive tomatoes [32]. Also, both F_v_/F_m_ from dark-adapted leaves and the fluorescence decrease ratio from illuminated leaves (R_FD_), that were related to photosystem integrity, can be used to discriminate cold tolerant from cold sensitive accessions of *A. thaliana* [23].

In the perspective of phenotyping the plant response to various stresses, a recurring question is to identify which CF parameters are impacted in response to stresses, thus providing a contrast that can be used to monitor the plant response to stresses. Such contrasted CF parameters may also allow the quantification of the plant resistance to stresses and therefore is of high interest for breeders [5]. Most studies only focused on the few CF parameters with known biological significance. Many other CF parameters can be measured or calculated even though their biological significance may remain obscure ([28,30]; Table 1). However, these could be useful in a phenotyping perspective using image analysis.

**Table 1:** **CF parameters used in this study**

**Symbol**	**Name** ^**§**^	**Formula**	**Col-0 control vs. Ler control**	**Col-0 inoculated vs. Col-0 control**	**Ler inoculated vs. Ler control**
F_O_	Minimal chlorophyll fluorescence intensity	Measured			
	measured in the dark-adapted state measured in the dark-adapted state (F_O_) and during the dark relaxation (FO_(85)_)				
F’_O_	Minimal chlorophyll fluorescence intensity measured in the light-adapted state	Measured			
	measured during the light adaptation (F’_O(n)_) and at the steady-state (F’_O(74)_)				
F_m_	Maximal chlorophyll fluorescence intensity measured in the dark-adapted state	Measured	_Fm(85)_*		
	measured in the dark-adapted state (F_m_) and during the dark adaptation (Fm_(85)_)				
F’_m_	Maximum chlorophyll fluorescence intensity measured in the light adapted state	Measured	F’_m(25)_**, F’_m(38)_**, F’_m(50)_*, F’_m(62)_*, F’_m(74)_*		
	measured during the ligth-adaptation (F’_m(n)_) and at the steady-state (F’_m(74)_)				
F_P_	Peak fluorescence during the initial phase of the Kautsky effect	Measured			
	measured at the begining of the light adaptation				
F_T_	Instantanueous fluorescence	Measured		F_T(25)_*, F_T(50)_*, F_T(62)_**, F_T(74)_*	
	measured in the dark-adapted state (F_T(85)_), during the light adaptation (F_T(n)_) and at the steady-state (F_T(74)_)				
F_v_/F_m_	Maximum PSII quantum yield	(F_m_-F_O_)/Fm	F_v_/F_m_**	F_v_/F_m_*	
	measured at the dark-adaptated state				
F’_v_/F’_m_	PSII quantum yield of light adapted sample	(F’_m_-F’_O_)/F’_m_	F’_v_/F’_m(25)_**, F’_v_/F’_m(38)_**, F’_v_/F’_m(50)_**, F’_v_/F’_m(62)_**, F’_v_/F’_m(74)_**		
	calculated during the light adaptation (F’_v_/F’_m(n)_) and at the steady-state (F’_v_/F’_m(74)_)				
NPQ	Non-photochemical quenching	(F_m_-F’_m_)/F_m_	NPQ_(38)_*	NPQ_(25)_*	NPQ_(85)_*, NPQ_(62)_**, NPQ_(74)_*
	calculated in the dark-adapted state (NPQ_(85)_during the light adaptation (NPQ_(n)_) and at the steady-state (NPQ_(74)_)				
qL	estimator of the fraction of open PSII centers	qP(F’_O_/F_T_)	qL_(25)_**, qL_(38)_**, qL_(50)_**, qL_(62)_**, qL_(74)_**		
	calculated during the light adaptation (qL_(n)_) and at the steady-state (qL_(74)_)				
qP	Coefficient of photochemical quenching	(F_m_-F_T_)/(F_m_-F_O_)	qP_(85)_**, qP_(25)_**, qP_(38)_**, qP_(50)_**,qP_(62)_*	qP_(25)_**, qP_(38)_**, qP_(50)_**,qP_(62)_**,qP_(74)_**	qP_(74)_*
	calculated in the dark-adapted state (qP_(85)_), during the light adaptation (qP(n)) and at the steady-state (qP_(74)_)				
Q_y_	Instantaneous PSII quantum yield	(F_m_-F_t_)/F_m_	Qy_(85)_**, Qy_(25)_*,Qy_(38)_**, Qy_(50)_*, Qy_(62)_*, Qy_(74)_*	Q_y(25)_**,Q_y(38)_**, Q_y(50)_**, Q_y(62)_**, Q_y(74)_**	
	calculated during the light adaptation (Qy_(n)_) and at the steady-state (Qy_(74)_)				
R_FD_	Fluorescence decline ratio	(F_p_-F_T_)/F_T_	R_FD(25)_**, R_FD(38)_**, R_FD(50)_**, R_FD(62)_**, R_FD(74)_*		R_FD(50)_**, R_FD(62)_**, R_FD(74)_**
	calculated during the light adaptation (R_FD(n)_) and at the steady-state (R_FD(74)_)				

Plants of ecotypes Ler and Col-0 of *A. thaliana* non-inoculated or inoculated with *P. ramosa* were imaged for 13 CF parameters (55 transient measures). This table presents the symbols and the names of the measured and the calculated CF parameters (^§^: n can be 25, 38, 50 or 62. For example, F_o_ was measured four times during the light adaptation at 25 s, 38 s, 50 s and 62 s).

Significant differences between the global CF values of non-inoculated and inoculated plants of ecotype Col-0, non-inoculated and inoculated plants of ecotype Ler or non-inoculated plants of ecotype Col-0 and non-inoculated plants of ecotype Ler are indicated respectively in the fourth, fifth and sixth columns (*: Mann–Whitney *U* test, p-value < 0.05, **: Mann–Whitney *U* test, p-value < 0.01).

Among the numerous pests impacting photosynthesis of the affected plants, the parasitic plants are atypical examples [33-35]. Some of them, including the broomrapes (*Orobanche* spp and *Phelipanche* spp), are harmful parasitic weeds causing devastating yield reductions in many important crops throughout the world. *Phelipanche ramosa* is by far the most widespread broomrape species [36]. *P. ramosa* depends on its host for all its nutrients and water supply, acquired from the host phloem and its life cycle has been well described in regard to its major host plants [37]. After the induction of seed germination by molecules exuded in the rhizosphere by host roots, a radicle emerges from the seed and attaches to the host root surface. The parasitic phase starts with the penetration of the parasite radicle into the host root through a differentiating haustorium, which connects to the host vascular tissues and serves as an attaching organ and as a bridge for water and nutrient transfer from the host. The parasite develops a tubercle, which gives rise to a subterranean shoot and then, after emergence from the soil, a branched flowering spike.

Success in stopping increased infestations needs a reliable strategy of integrated management [38,39], where breeding resistant crops should be one of the key elements. In this context and since host derived resistance remains rare, large scale screenings of genetic resources are of main importance for crop breeders. Classical descriptors for host resistance against broomrapes today mainly consist in the assessment of the number of emerged flowering spikes during field trials [40,41], and in the assessment of the number of *P. ramosa* attachments to host roots as well as of the kinetics of infection in pots or mini-rhizotrons experiments [42,43]. The measure of these classical descriptors remains time consuming and unsuitable for large scale and high-throughput screenings.

Therefore, the development of high-throughput phenotyping system based on images is of major interest as it would be a dramatic improvement in the screening process. Root infection by *P. ramosa* has a systemic impact that could probably be observed on leaves. The identification of such a foliar phenotype may greatly facilitate phenotyping of the response of *A. thaliana* to *P. ramosa*. Thus, in this paper, we aimed at identifying CF parameters impacted during the infection of *A. thaliana* by *P. ramosa*. We measured and calculated various CF parameters (Table 1) on the two ecotypes Columbia-0 (Col-0) and Landsberg (Ler) of *A. thaliana* non-inoculated vs inoculated with *P. ramosa*. For each plant, 55 images and their associated histograms were recorded. We developed a script in the R software aiming at facilitating the identification of CF parameters impacted during the infection. We implemented this R script as a web application at http://www.phenoplant.org. The R script described in the present paper was associated to a script previously described [5] in order to provide easy-to-use web resources for the analysis of CF imaging datasets.

## Results

**Figure 1:**
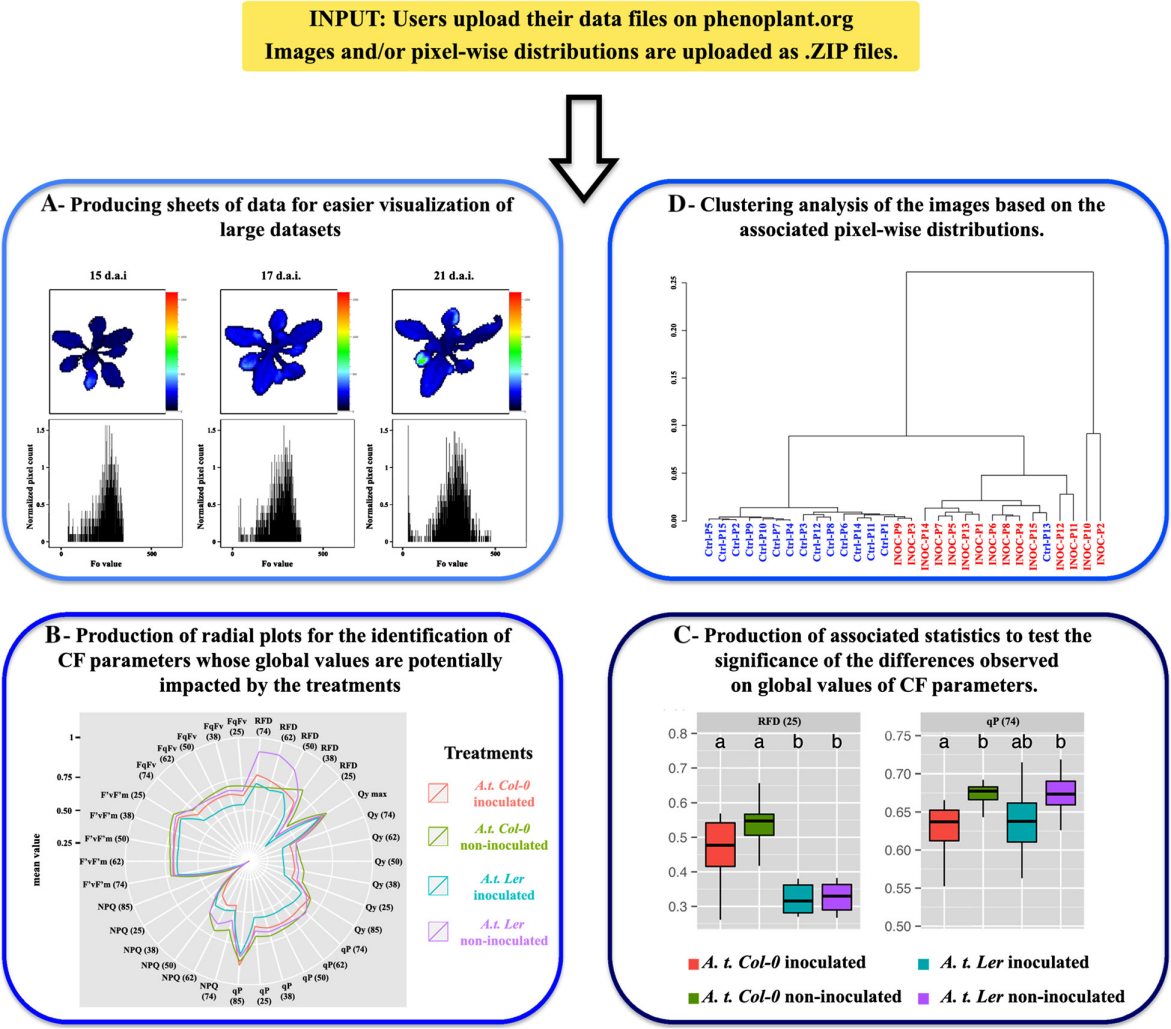
**Work flow of the R script.** The users upload the previously captured fluorescence images and/or their associated histograms as input. The data are sorted out according to the information indicated by the user on the web interface. To get an overview of the data, the data are represented in the form of contact sheets of images or of histograms **(A)**. Here, *A. thaliana* plants were imaged 15, 17 and 21 days after inoculation (d.a.i.) by *P. ramosa*. Images and histograms are displayed on the contact sheet to visualize a variation during time. The mean of the global CF values for each treatment are graphically represented in a radial plot where each point represents the mean for one treatment and each radius represents a CF parameter **(B)**. In order to detect a significant difference in the global CF values between the treatments, the means of the global CF values for each treatment are represented in the form of a box plot **(C)** in which horizontal lines indicate the 0.25 and 0.75 percentile from bottom to top, the interior line indicates the median and the exterior shapes represent the overall distribution. The results of the Mann–Whitney U tests are indicated in box plots. To compare the various histograms, a dendrogram based on the histograms is produced **(D)**.

To describe the impact of *P. ramosa* on the photosynthetic performance of *A. thaliana*, CF parameters were compared: inoculated vs. non-inoculated plants of ecotypes Col-0 and Ler. CF parameters were measured or calculated at the dark-adapted state, during the light-adaptation, at the light-adapted steady-state and during the dark-adaptation (Table 1). Finally, every measuring day, 55 images were recorded for each plant. Given our experimental design, our dataset reached 9900 image files and 9900 text files containing the histograms associated to each image. Such a dataset is too big to be manually analyzed. Therefore, we aimed at developing a script that first, automatically sorts out the data to provide an easy overview of the data; second, facilitates the identification of CF parameters significantly impacted during infection of *A. thaliana* by *P. ramosa* (Figure 1); and third, is available as an interface to the plant phenotyping community.

### Overview of the dataset by creating contact sheets of images and associated histograms

The aim of such an overview of the data is to make the visual inspection of all the images and associated histograms easier. Therefore, the script sorts out the images and/or the histograms on three types of contact sheets (Figure 1A).

First, to control the quality of images, each image faces its associated histogram. One contact sheet per plant and per CF parameter is produced. Second, contact sheets are produced that enable the comparison of the images (or histograms) of plants having undergone different treatments. Thus, on these contact sheets, all the images (or histograms) from one treatment face those from another treatment. These sheets enable the rapid visual identification of CF parameter(s) potentially impacted by a treatment. For example, the rapid visual inspection of such contact sheets suggests that the coefficient of photochemical quenching during light adaptation (qP) is potentially impacted by the infection by *P. ramosa*. Indeed, *A. thaliana* plants ecotype Col-0 inoculated with *P. ramosa* display values of qP lower than non-inoculated plants (Additional file 1).

Finally, contact sheets are produced that enable the visual inspection of the potential intensity dynamics of CF parameters over time. These contact sheets display the kinetics of images (or histograms) for one transient measure on the same plant over the duration of the monitoring. Such contact sheets allow the identification of CF parameters declining over time due to the inoculation with *P. ramosa*. For example, qP declined for plants of ecotype Col-0 inoculated with *P. ramosa*, contrary to non-inoculated plants (Additional file 2).

### Detection of CF parameters impacted by a treatment using global values

Global fluorescence values, i.e. the mean of the values of all the pixels in the image, are well suited for a rapid comparison of the impact of *P. ramosa* on the CF parameters. First, we plotted the global fluorescence values of all the CF transient measures for each treatment on a same radial plot (Figure 1B). Such a plot provides a synthetic view of the comparisons between treatments for all transient measures. The observation of the radial plots highlighted the variation of various transient measures, apparently due to the infection of *A. thaliana* ecotypes Col-0 and Ler by *P. ramosa*. Interestingly, several transient measures may also highlight differences between non-inoculated plants of Col-0 and Ler ecotypes.

In order to test whether these variations are significant, a detailed view of the comparisons between treatments is provided for each transient measure in the form of box plots (Figure 1C). The box plots display more information than the radial plots as minimum and maximum values observed, lower and upper quartiles and percentiles are represented for each transient measure. Moreover, the significance of the differences observed is tested using the Mann–Whitney *U* test [44]. Such a detailed view shows that several CF parameters vary significantly between the various conditions studied.

First, we compared non-inoculated plants from Col-0 and Ler ecotypes and identified CF parameters that could discriminate between these ecotypes at 21 days after inoculation (d.a.i.). Eight CF parameters presented global fluorescence values significantly different among non-inoculated plants of the ecotypes Col-0 and Ler (Mann–Whitney *U* test, p-value < 0.05; Table 1): the maximal chlorophyll fluorescence intensity in light (F’_m_) and in dark-adaptation (F_m(85)_), the maximum PSII quantum yield (F_v_/F_m_), the PSII quantum yield in light (F’_v_/F’_m_), the instantaneous non-photochemical quenching during the light-adaptation (NPQ), the estimator of the fraction of open PSII centers (qL), the coefficient of photochemical quenching (qP), the instantaneous PSII quantum yield (Q_y_) and the instantaneous fluorescence decline ratio in light (R_FD_).

Second, five CF parameters altered by the infection by *P. ramosa* were identified for the two ecotypes at 21 d.a.i. (Mann–Whitney *U* test, p-value < 0.05; Table 1). For ecotype Ler, NPQ in light and during dark-adaptation, qP in steady-state and R_FD_ in light were significantly lower among plants inoculated with *P. ramosa* and non-inoculated plants. For ecotype Col-0, the instantaneous fluorescence (F_T_), NPQ in light, qP and Q_y_ in light are significantly negatively impacted by the infection.

### Discriminating between treatments using a clustering analysis based on the histograms

Results based on the global values provided good candidate CF parameters meant to be impacted by the infection by *P. ramosa*. However, images contain far more information compared to the global values. Therefore, for each CF transient measures, we sorted out all the images by performing a clustering approach on histograms. For this, we calculated distances between images using the Bhattacharyya coefficient [45]. Then, we used six available agglomeration methods to construct dendrograms (Figure 1D): single linkage, complete linkage, UPGMA, WPGMA, WPGMC and UPGMC.

In most cases, the dendrograms obtained with single linkage, WPGMC and UPGMC agglomeration methods did not cluster on a same branch either images of plants having undergone the same treatments, or plants sharing a similar apparent health status (Additional file 3). Alternatively, using the WPGMA, complete linkage and UPGMA agglomeration methods, images clustered on a same branch in the dendrograms corresponded to plants sharing a same treatment or a same apparent health status. Some images however were mis-clustered regarding either the treatments or the health status of plants. Rates of mis-clustered images are reported in Additional file 3.

**Figure 2:**
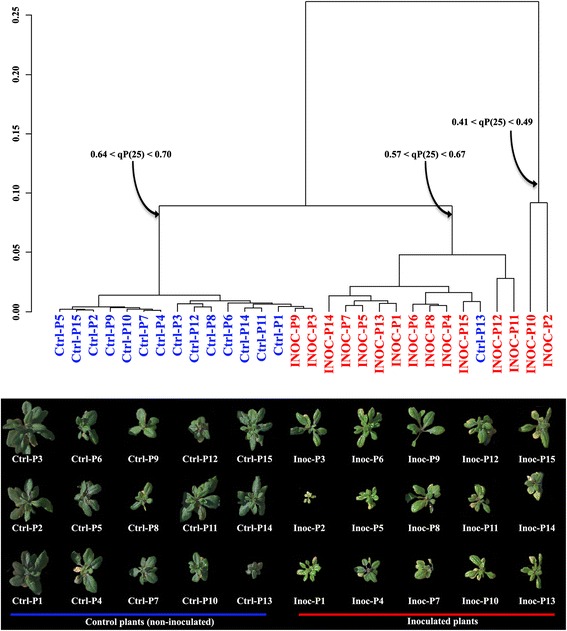
**Clustering analysis of plants of ecotype Col-0 non-inoculated or inoculated with** ***P*** **.** ***ramosa*** **.** Fifteen plants of *A. thaliana* of ecotype Col-0 were inoculated with *P. ramosa* and 15 plants were not inoculated. The plants were imaged for 55 fluorescence parameters. Dendrograms were built based on the histograms of the CF parameter qP_(25)_ using the WPGMA agglomeration method. Inoculated plants (red) and non-inoculated plants (blue) globally belong to different branches of the dendrogram. However, inoculated plants INOC-P9 and INOC-P3 group with non-inoculated plants. These plants are visually healthier than other inoculated plants, as seen on photo at the bottom of the figure. The non-inoculated plant Ctrl-P13 groups with inoculated plants. This plant is visually less healthy than other non-inoculated plants.

Among the candidate CF parameters identified for plants of ecotype Col-0 using global values, qP measured during the light-adaptation (qP_(25)_, qP_(38)_, qP_(62)_ and qP_(74)_) and Q_y_ measured in the beginning of the light-adaptation (Q_y(25)_ and Q_y(38)_) allowed clustering of the images according to the inoculation status of plants. Using the agglomeration WPGMA, complete linkage or UPGMA agglomeration methods for images obtained with these CF transient measures, we observed an average of 20% of mis-clustering when considering the plant treatment (Additional file 3). However, when considering the apparent health status of plants as defined by visual observation of plants by experts, less than 10% of the images appeared mis-clustered on the dendrograms (Additional file 3). For example, for the CF parameter qP_(25)_, two major clusters can be observed on the dendrogram (Figure 2). Images of all non-inoculated plants except one clustered with images of two inoculated plants to form a first major cluster. The inoculated plants corresponding to the two mis-clustered images in this first cluster appeared in much better health than the other inoculated plants (Figure 2). Infection level was checked for these plants. Macroscopic observations confirmed that inoculation failed, thus explaining their position in the dendrogram. The second major cluster is composed by inoculated plants and one non-inoculated plant. The growth of the mis-clustered non-inoculated plant was strongly altered, suggesting that this plant had undergone a heavy stress (Figure 2). For plants of ecotype Ler, results obtained with the clustering approach are less contrasted for any of the candidate CF parameter.

*P. ramosa* attachments to host roots does not result in clearly delimited eye-visible symptoms on *A. thaliana* leaves. Therefore, in order to further check the correct sorting out of the CF images by the clustering procedure, we used a set of images of bean leaves inoculated with the plant pathogenic bacterium *Xanthomonas fuscans* subsp. *fuscans*, previously described [5]. In contrast to *A. thaliana* infected by *P. ramosa*, leaves in these pictures displayed clearly recognizable symptoms of common bacterial blight. On bean leaves, lesions are clearly delimited and visible to the eye, which enables to control that the CF parameter displays contrasts between impacted and healthy plant tissues. Most healthy control plants clustered together, whereas most inoculated plants formed a separate cluster (Additional file 4). Using this dataset as well, we could explain all the apparent mis-clustering events: inoculated leaves that clustered with non-inoculated ones corresponded to leaves for which inoculation failed. Moreover, non-inoculated leaves that clustered with inoculated ones corresponded to leaves that displayed obvious stresses or growth defects (Additional file 5). The second advantage using a bean image dataset is that we can quantify the amount of symptoms. We previously showed that the CF parameter F_v_/F_m_ could actually be reliable to quantify the amounts of symptoms on bean leaves [5]. Interestingly, in this dataset, the obtained dendrogram clustered images according to their respective amounts of symptoms (Additional file 5).

### A web application available at http://www.phenoplant.org

In order to make it available to the plant phenotyping community, the procedure described in the present paper was implemented in a web resource available at http://www.phenoplant.org. The web resource gathers two scripts aiming at making the analysis of fluorescence images easier. We also implemented a second script previously described aiming at the quantitative phenotyping of plant resistance to a biotic and abiotic stresses [5]. The source files are available for download at http://www.phenoplant.org.

Users may upload their datasets as zip files on the web site, and may select their preferences among the proposed options for the analysis. It was designed for images of individual leaves or plants and it accommodates images in TIFF format and histograms in text format. Once the analysis is done, users receive an e-mail indicating where to retrieve the results of the analysis. Results are returned in the portable form of PDF and Excel files.

## Discussion

We propose a web resource devoted to the image analysis of large CF image datasets. This resource was designed for CF image datasets but could probably be adapted also to other types of image dataset, if relevant. This resource requires grayscale images in TIFF format and their associated histograms. Datasets are uploaded at http://www.phenoplant.org as zip files. Users just have to follow a specific nomenclature for image files or folders that is explained in the user’s guidelines.

This application contains two types of image analysis procedures that answer two different questions. A previously described script aims at the quantitative phenotyping of plant resistance to biotic and abiotic stresses [5]. Quantitative phenotyping is important in plant breeding in order, for example to screen accessions for resistance to pathogens as plant resistance to pathogens may either be qualitative or quantitative. Thus, when a given CF parameter can be used as a descriptor of plant stress, the quantitative procedure implemented in this script enables the evaluation of the severity of the stress.

In the present paper, a new script is described to provide three complementary approaches to get an overview of large image datasets, and to identify CF parameters that can be used as descriptors of plant stresses. Several other algorithms are described in the literature to perform specific image analyses such as the measuring of the area of leaves, plants or grains [12-14], the impact of pests and abiotic stresses [5,12,13] or the callose deposition [15]. Free web-based applications such as PhenoPhyte [12] are available to the plant phenotyping community to analyze plant images obtained by conventional imaging techniques. However, to our knowledge, no software is providing an overview of large image datasets. Given the size expected for high-throughput phenotyping datasets, even simple procedures are problematic if not automated. In our view, a simple algorithm that sorts out the images to provide relevant contact sheets will help the user to get acquainted with his data. Therefore, the first output by the script described in the present paper produces contact sheets of images or histograms meant to visually compare plants having undergone different treatments.

The second output of the script aims at the identification of transient measures that significantly discriminate between two groups of plants (i.e. Col-0 vs. Ler plants, inoculated vs. non-inoculated plants, healthy vs. stressed plants). Most CF parameter measures could significantly discriminate between ecotypes Col-0 and Ler (Table 1). Therefore, CF imaging can thus be used to discriminate between ecotypes of *A. thaliana*. Such a discrimination between plant genotypes was already proposed in a previous study on the use of CF imaging for the discrimination between plant species from the genus *Origanum* [46].

We then compared the global CF values obtained for plants having undergone different treatments. Many studies reported the interest of using CF imaging to monitor the impact of biotic and non biotic stresses on plant tissues [18,21]. In the case of the infection of *A. thaliana* by *P. ramosa*, we identified five CF parameters as potential markers of *A. thaliana* stress by *P. ramosa*. Interestingly, Col-0 and Ler ecotypes seemed to respond differently to *P. ramosa*, as CF parameters impacted by the infection differ from one ecotype to another (Table 1). To our knowledge, there is no study about such a differential response of *A. thaliana* to a biotic stress. However, this shows that for each plant or each ecotype, an initial analysis aiming at identifying a good CF parameter candidate is necessary prior to quantifying the impact of infection on the plant physiology. Future studies should involve the screening of a large number of ecotypes, in order to select relevant CF parameters to use for screening for resistance against *P. ramosa*. Relevant CF parameters should be impacted on most sensitive ecotypes and such an impact should depict the severity of the disease by *P. ramosa*. Moreover, for each crop of interest (e.g. sunflower or rape), similar screenings should be performed on many varieties to identify what relevant parameters to use for phenotyping. According to the results of the present study, the CF parameter qP of light-adapted leaves may constitute a good candidate. Interestingly in some cases, variations of CF parameters may be recorded prior to the occurrence of eye-visible symptoms [5,26,27], thereby greatly speeding up the process for the selection of resistant crop varieties.

The diversity of CF parameters impacted among various ecotypes of *A. thaliana* during the infection of *A. thaliana* by *P. ramosa* may represent various types of response to the infection. Actually, after the infection by *P. ramosa*, the ecotype Col-0 of *A. thaliana* displayed clearly visible symptoms of growth defect, chlorosis and in some cases marginal necrosis. For the ecotype Ler, no clear visible symptoms were observed in our experiments, despite the fact that attachment to roots and infection were successful (data not shown). Although we do not have comprehensive data on the behavior of ecotype Ler of *A. thaliana*, such observations suggest that this ecotype may be tolerant to the infection by *P. ramosa*. Future studies should help establish whether the different CF parameters impacted in the Col-0 or Ler situations may be used to screen either for sensitivity or tolerance to *P. ramosa*. Nonetheless, it is interesting to note that among the CF parameters impacted by the infection, a significant decrease of R_FD_ occurred in the ecotype Ler that is potentially tolerant, but not in ecotype Col-0 that is sensitive to *P. ramosa*. In response to cold treatment, on the contrary, cold tolerant accessions such as Col-0 displayed a smaller decrease of R_FD_ compared to sensitive or intermediate accessions such as Ler [23]. The identification of CF parameters impacted by the infection may provide interesting hypotheses for studying the mechanisms of interaction between *A. thaliana* and *P. ramosa*. A decrease of qP reveals a decrease in the proportion of open reaction centers, *i.e*. in the proportion of reaction centers that are able to accept further electrons [30]. So with Col-0, the observed decrease of qP following the infection by *P. ramosa* shows that the reoxydation of quinones during photosynthesis is altered. As R_FD_ is directly correlated to the net CO_2_ assimilation rate [47], a decrease in R_FD_ may reveal a decrease in CO_2_ assimilation in response to the infection by *P. ramosa*.

In a third approach, we intended to better take into account the information contained in the images. For this purpose, the entire histograms of each image were compared. Indeed, shapes of histograms differ on images of stressed plants compared to healthy plants [5,16]. Here, we proposed to discriminate between treatments following the assumption that the distance between the histograms of plants under different treatments is higher than between plants under the same treatment. A similar clustering approach was recently described for the automated sorting of fluorescence microscopy images [48]. Using this procedure, we could retrieve images of plants giving false negative or false positive results, i.e. inoculated plants for which inoculation failed or control plants that were stressed in an uncontrolled manner, respectively. Experiments conducted on the control dataset of bean images suggest that such a clustering approach may allow a grouping of images displaying similar stress intensity. In the present paper, such a classification procedure was based on images obtained from single measures of CF parameter. The use of such a clustering approach on images that would have been reconstructed through a combinatorial imaging approach [11,23,32,49] may reveal a very powerful tool for an automated classification of plants according to their health status.

## Conclusions

In this paper, we describe a web resource available at http://www.phenoplant.org, devoted to the image analysis of large CF image datasets. We propose two types of image analysis. A first analysis aims at the quantitative phenotyping of plant resistance to a stress, as described in [5]. A second analysis, described in this paper, aims at providing an overview of large image datasets and at the identification of CF parameters impacted by a stress. On the one hand, CF parameters of interest are first identified based on a comparison of their global values (i.e. the mean value of the parameter over the whole imaged plant). On the other hand, CF parameters of interest may be identified using a clustering approach based on pairwise distances between images. In the latter, each pixel-value is taken into account, in an attempt to exploit maximum information in each image. Such an analysis allows both the identification of CF parameters impacted by a stress and the detection of unhealthy plants or leaves.

## Methods

### Biological material

*P. ramosa* (L.) Pomel seeds were collected from mature flowering spikes on boomrape parasitized oilseed rape field (*Brassica napus*) in Saint-Jean-d’Angély (France) in 2012, and stored at 25°C in darkness until use. Seeds were surface-sterilized for 5 min in sodium hypochlorite (12%), and thoroughly rinsed three times for 1 min and three times for 5 min with sterile distilled water. Seeds were then suspended in conditioning medium containing 1.10^−3^ M Na/K phosphate buffer (pH 7.5, adjusted with KOH) and PPM 0,1% (Plant Preservative Mixture, Kalys, Bernin, France), with a ratio of 10 mg seeds.mL^−1^. Seeds were then placed in the dark at 21°C for 7 days for conditioning. The conditioned seeds were stimulated by adding the synthetic strigolactone GR24 (germination stimulant) at a final concentration of 10^−9^ M in 0.1% v/v acetone. GR24 treatments were carried out at 21°C in the dark for 48 hours and subsequently used for *A. thaliana* root inoculation.

For co-cultivation experiments, *A. thaliana* seeds (Columbia-0 (Col-0) and Landsberg (Ler) ecotypes) were surface sterilized in a closed container with chlorine gas for 3 h (http://www.plantpath.wisc.edu/fac/afb/vapster.html). Surface-sterilized seeds were transferred to 9.4 cm Ø plates containing MS medium supplemented with 0.6% w/v agar. Following stratification for 3 days at 4°C, seeds were incubated at 21°C in a growth chamber (16 h light, 8 h dark) for 15 days. Seedlings were then transferred to 15 cm Ø plates containing MS medium supplemented with 0.6% w/v agar. Plates were incubated vertically at 21°C in a growth chamber (16 h light, 8 h dark) for 7 days. Plantlets (3 per plate) were then transferred onto filter papers each covered or not with 20 mg of GR24 treated *P. ramosa* seeds placed in cut 12 × 12 cm square plates containing a uniform layer of rockwool moisturized with 50 mL of 0.5× Tadano and Tanaka growth medium Tadano and Tanaka, 1980. Five plates per condition (inoculated or not) were used giving a total of 15 non-inoculated plants and 15 plants inoculated with *P. ramosa*. Plates were incubated vertically at 21°C in a growth chamber (16 h light, 8 h dark, 70% humidity) for 15 days and watered every 2 days with 10 mL TT medium.

A second set was used to test the relevance of the method described and was composed by 40 plants of bean (*Phaseolus vulgaris*, cv. Flavert). Twenty plants were mock-inoculated and twenty plants were inoculated with *Xanthomonas fuscans* subsp. *fuscans* strain CFBP4834-R. Technical details are given in [5].

### Technical setup and image acquisition

The PSI Open FluorCam FC 800-O (PSI, Brno, Czech Republic) was used to image plants of ecotypes Col-0 and Ler of *A. thaliana*. Both non-inoculated plants and plants inoculated with *P. ramosa* were imaged. The plants were imaged 15, 17 and 21 days after inoculation (d.a.i.). The system sensor is a CCD camera with a pixel resolution of 512 by 512 and a 12-bit dynamic. The system includes four LED panels divided in two pairs. One pair provides actinic light in orange wavelength of around 618 mm, with an intensity that can vary from 200 to 400 μmol/m^2^/s. The other pair provides a saturating pulse during 1 s in blue wavelength, typically 455 mm, with an intensity of up to 3000 μmol/m^2^/s.

Plants were dark adapted for 45 minutes before taking image series. At the beginning of the light protocol, the minimum fluorescence (F_O_) was measured. A saturating pulse allowed the measuring of the maximum fluorescence (F_m_) and was followed by a dark relaxation period of 10 s. Then, an actinic light (230 μmol/m^2^/s) was applied during 60 s. At the beginning of the application of the actinic light (at 16 s), the fluorescence peak during the initial phase of the Kautsky effect (F_p_) was measured. At 25, 38, 50, 62 and 74 s, during the application of the actinic light, the instantaneous fluorescence (F_T_) was measured, a saturating pulse was applied to measure the maximum fluorescence at light (F’_m_) and a short exposure to far-red irradiance allows the measuring of the minimum fluorescence at light (F’_O_) during the light adaptation and at the steady-state (74 s). The actinic light period was followed by a dark relaxation period of 20 s. At 85 s, F_O_, F_m_ and F_T_ were measured in a dark relaxation state.

Some images come from the equation of measured parameters [50-54]: the PSII quantum yield of light-adapted plants in light or in steady state (F’_v_/F’_m_), the instantaneous non-photochemical quenching in light during dark relaxation or light adaptation or at the steady-state (NPQ), the coefficient of photochemical quenching of variable fluorescence based on the lake model of PSII in light or in steady-state (qL), the coefficient of photochemical quenching of variable fluorescence based on the puddle model of PSII during dark relaxation, light adaptation or at steady state (qP), the instantaneous PSII quantum yield during dark relaxation, light adaptation and in the steady state (Q_y_), the maximum PSII quantum yield (Q_y_max_), the instantaneous fluorescence decline ratio in light and at the steady-state (R_FD_).

Thus, for each plant, 55 images and their associated histogram were produced during the protocole, giving 9900 images and 9900 histograms (2 ecotypes of *A. thaliana* × 15 plants × 2 treatments × 3 days × 55 CF transient measures). A figure expliciting the various CF parameters used in this study is available in the FluorCam7 user manual, page 9 (downloadable at http://www.psi.cz/downloads/).

### Creation of contact sheets of images and associated histograms

The application draws contact sheets of the images and the histograms. First, the images and histograms are sorted according to the treatment made on the plants. Then, the images are read and displayed using the R package EBImage [55]. The histograms are displayed using the R package ggplot2 [45]. One contact sheet per parameter, per treatment and per day of measurement is returned.

### Comparison of the global values of the CF parameters

For each image, the mean of the values of all the pixels is calculated to obtain the global value of the image. Then, for each treatment the mean of the global values is represented on radial plots using the R package ggplot2 [45]. Four radial plots are drawn separately to display parameters whose mean is less than 1, between 1 and 25, between 25 and 300, and superior to 300. Box plots describing the minimum and maximum values observed, the lower and upper quartile and the percentile are drawn using the R package ggplot2 [45]. A Mann–Whitney *U* test [44] is performed using the wilcox.test function implemented in R to statistically compare the global CF values observed for each treatment.

### Clustering based on the histograms

The script builds dendrograms in which each leaf represents a histogram. First, the distances between all the pairs of histograms were calculated from the Bhattacharyya coefficient [45] using the R package StatMatch [56]. Second, a clustering step is performed according to six agglomeration methods, *i.e*. single linkage (“single”), complete linkage (“complete”), UPGMA (“average”), WPGMA (“mcquitty”), WPGMC (“median”) and UPGMC (“centroid”) using the function hclust of the R package stats [57]. Finally, a dendrogram is calculated based on the clustering step, using the R package stats [57].

The relevance of the dendrograms produced through the various agglomeration methods was tested in relation to either the treatment of the plants (i.e. inoculated vs. non-inoculated), or according to their apparent health status. The apparent health had previously been estimated by experts after visual inspection of color photographs for each plant. For both treatment and apparent health, the rate of mis-clustered images was determined for each dendrogram obtained, using the various agglomeration methods.

Technical limitations of the www.phenoplant.org web service are mentioned on the website, and may improve overtime. At the present date, data upload is limited to 2048 Mo (2 Go). The script is implemented on a 16Go RAM server.

The dataset used in this paper gives a zip file of 114 Mo and the complete analysis run during 10 hours.

The source files for the R scripts are published on the web site under CeCILL FREE SOFTWARE LICENSE AGREEMENT, and may be downloaded at the following URL: http://www.phenoplant.org/#tdm8.

## Additional files

Additional file 1:
**Comparison of images of plants of** ***A. thaliana*** **ecotype Col-0 non-inoculated or inoculated with** ***P. ramosa. ***Plants of ecotypes Col-0 of *A. thaliana* non-inoculated or inoculated with *P. ramosa* were imaged for qP_(25)_ 21 days after inoculation (d.a.i.). Each color of the image represent a qP_(25)_ value. Non-inoculated plants display qP_(25)_ globally higher than inoculated plants. Indeed, on images of non-inoculated plants, most of the pixel correspond to qP_(25)_ values between 0.7 and 0.8. On images of inoculated plants, most of the pixel correspond to qP_(25)_ values between 0.55 and 0.65. Additional file 2:
**Dynamics of qP**_**(25)**_ **over time for plants of** ***A. thaliana*** **ecotype Col-0 inoculated with** ***P. ramosa.*** Plants of ecotypes Col-0 of *A. thaliana* inoculated with *P. ramosa* were imaged for qP_(25)_ 15, 17 and 21 days after inoculation (d.a.i.). Each color of the image represent a qP_(25)_ value. At 15 and 17 d.a.i., the plant displays qP_(25)_ values superior to 0.65. At 21 d.a.i., plant displays qP_(25)_ values inferior to 0.65. Additional file 3:
**Relevance of the dendrograms.** Various agglomeration methods (single linkage, WPGMC, WPGMA, complete linkage, UPGMA and UPGMC) were used to construct images based-dendrograms. The relevance was tested in relation to either the treatment of the plants (i.e. inoculated vs. non-inoculated), or according to their apparent health status. This table indicates the percentages of mis-clustered plants for each agglomeration method (nd: not discriminant). CF parameters presenting less than 10% of mis-clustering on the base of the apparent health of the plants are indicated in bold. Additional file 4:
**Robustness of the clustering procedure.** Bean leaflets of the cultivar Flavert inoculated with *X. fuscans* subsp. *fuscans* CFBP4834-R (1.10^6^ CFU.ml^−1^) or mock were imaged for the fluorescence parameter F_v_/F_m_. A dendrogram (on the left) based on the histograms was built from the UPGMC agglomeration method. Globally, CFBP4834-R-inoculated leaflets (red) and mock-inoculated leaflets (blue) belong to different groups. Two main clusters can be observed. (i) Images of most of the mock-inoculated leaflet and of the CFBP4834-R-inoculated leaflet 4834R-p3 form a first cluster. The histograms (on the right) of these images are similar and present a peak around the F_v_/F_m_ value of 0.8 (indicated by an orange dotted-line). (ii) Images of most of the CFBP4834R-inoculated leaflet and of the mock-inoculated leaflets H_2_O-p16, H_2_O-p6 and H_2_O-p12 form a second cluster. The histograms of these images are right-shifted with regard to those of the first cluster and can present different shapes. Additional file 5:
**Images of bean leaves cluster according to the severity of symptoms.** Bean leaflets of the cultivar Flavert inoculated with *X. fuscans* subsp. *fuscans* CFBP4834-R (1.10^6^ CFU.ml^−1^) or mock were imaged for the fluorescence parameter F_v_/F_m_. The figure represents an enlargement of the shaded part of the dendrogram found in Additional file 4. Five clusters of histograms can be defined according to the amount of symptoms (indicated by coloured boxes). The image of one leaflet representative of each group is displayed at the bottom. Low intensity pixels are displayed by high intensities of black and correspond to unhealthy tissues (the scale of intensities of F_v_/F_m_ is displayed on the right of the images). The leaflet 4834R-p5 forms a full cluster and displays 68.5% of symptoms. The grey box is composed by the histograms of leaflets displaying between 20.5 and 24.1% of symptoms. The purple box is composed by the histograms of leaflets displaying between 14.2 and 15.6% of symptoms. The leaflet 4834R-p11 forms a full cluster and displays 9.7% of symptoms. The orange box is composed by the histograms of leaflets displaying less than 6.2% of symptoms.
