# Supplementary figures and images for: Phenoplant: a web resource for the exploration of large chlorophyll fluorescence image datasets
Source: Plant Methods. 2015 Apr 3;11:24. doi: 10.1186/s13007-015-0068-4 (PMC4392743; doi:10.1186/s13007-015-0068-4)

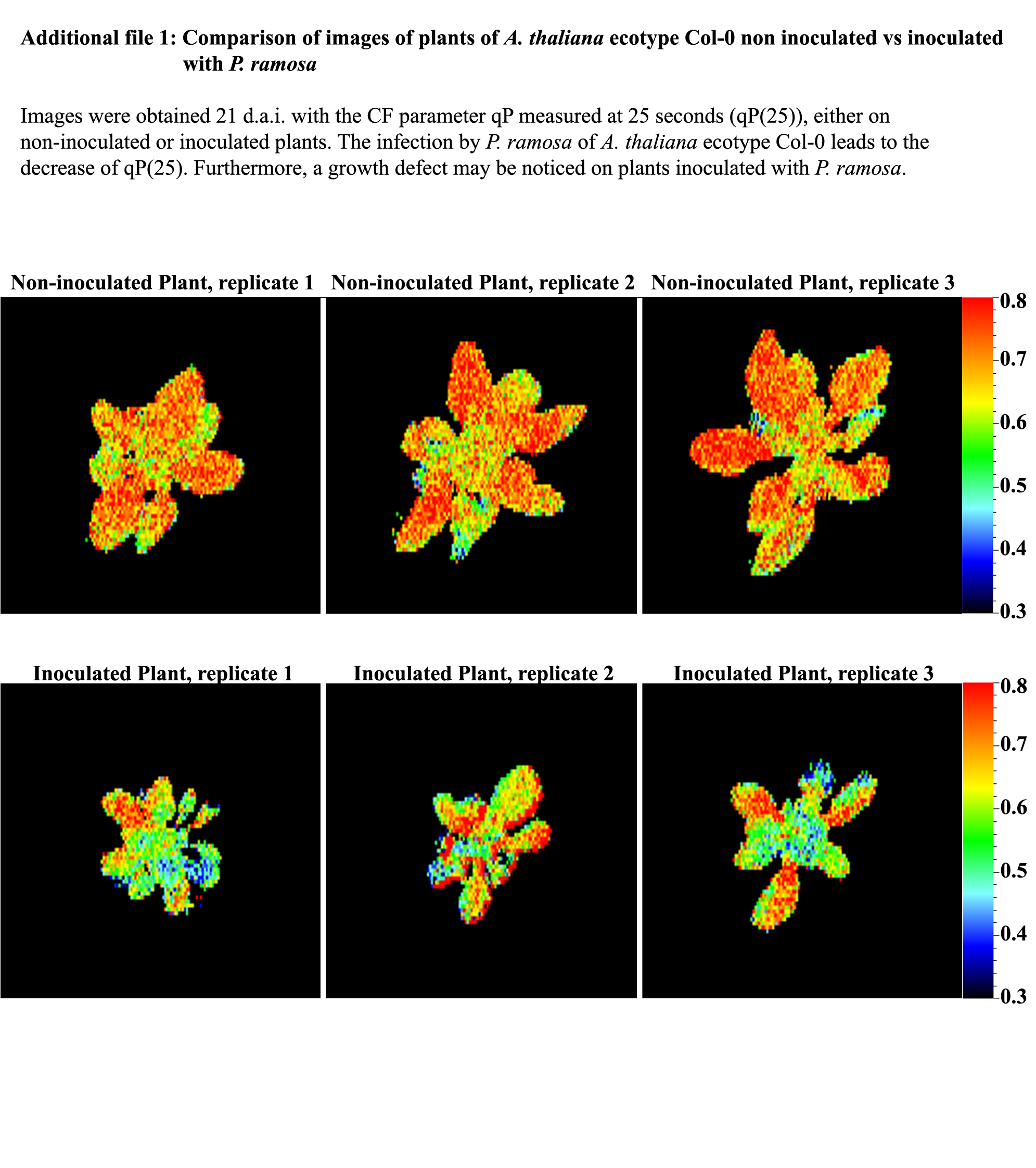

Supplement: Additional file 1: — Comparison of images of plants of A. thaliana ecotype Col-0 non-inoculated or inoculated with P. ramosa. Plants of ecotypes Col-0 of A. thaliana non-inoculated or inoculated with P. ramosa were imaged for qP(25) 21 days after inoculation (d.a.i.). Each color of the image represent a qP(25) value. Non-inoculated plants display qP(25) globally higher than inoculated plants. Indeed, on images of non-inoculated plants, most of the pixel correspond to qP(25) values between 0.7 and 0.8. On images of inoculated plants, most of the pixel correspond to qP(25) values between 0.55 and 0.65. [file 13007_2015_68_MOESM1_ESM.jpg]

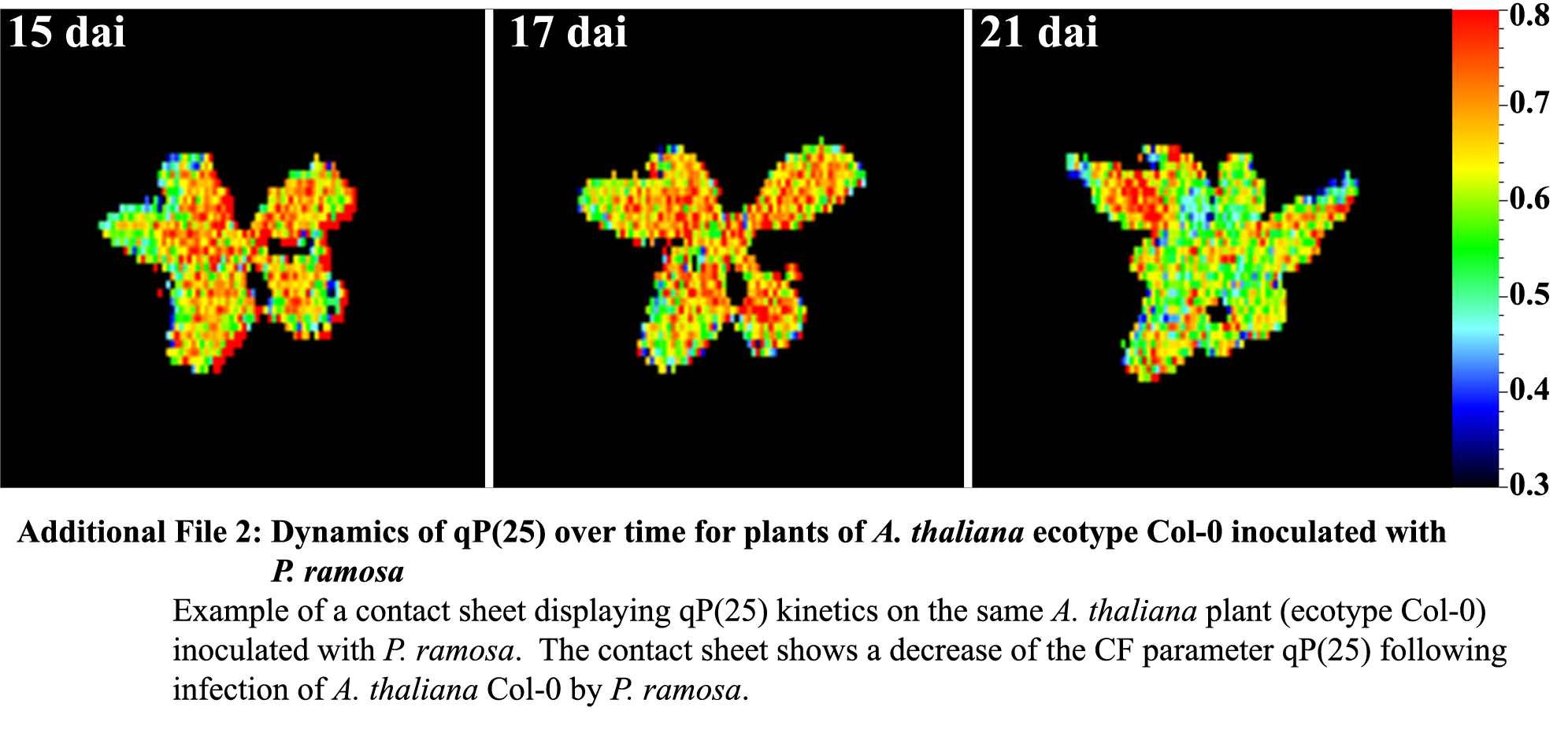

Supplement: Additional file 2: — Dynamics of qP (25) over time for plants of A. thaliana ecotype Col-0 inoculated with P. ramosa. Plants of ecotypes Col-0 of A. thaliana inoculated with P. ramosa were imaged for qP(25) 15, 17 and 21 days after inoculation (d.a.i.). Each color of the image represent a qP(25) value. At 15 and 17 d.a.i., the plant displays qP(25) values superior to 0.65. At 21 d.a.i., plant displays qP(25) values inferior to 0.65. [file 13007_2015_68_MOESM2_ESM.jpg]

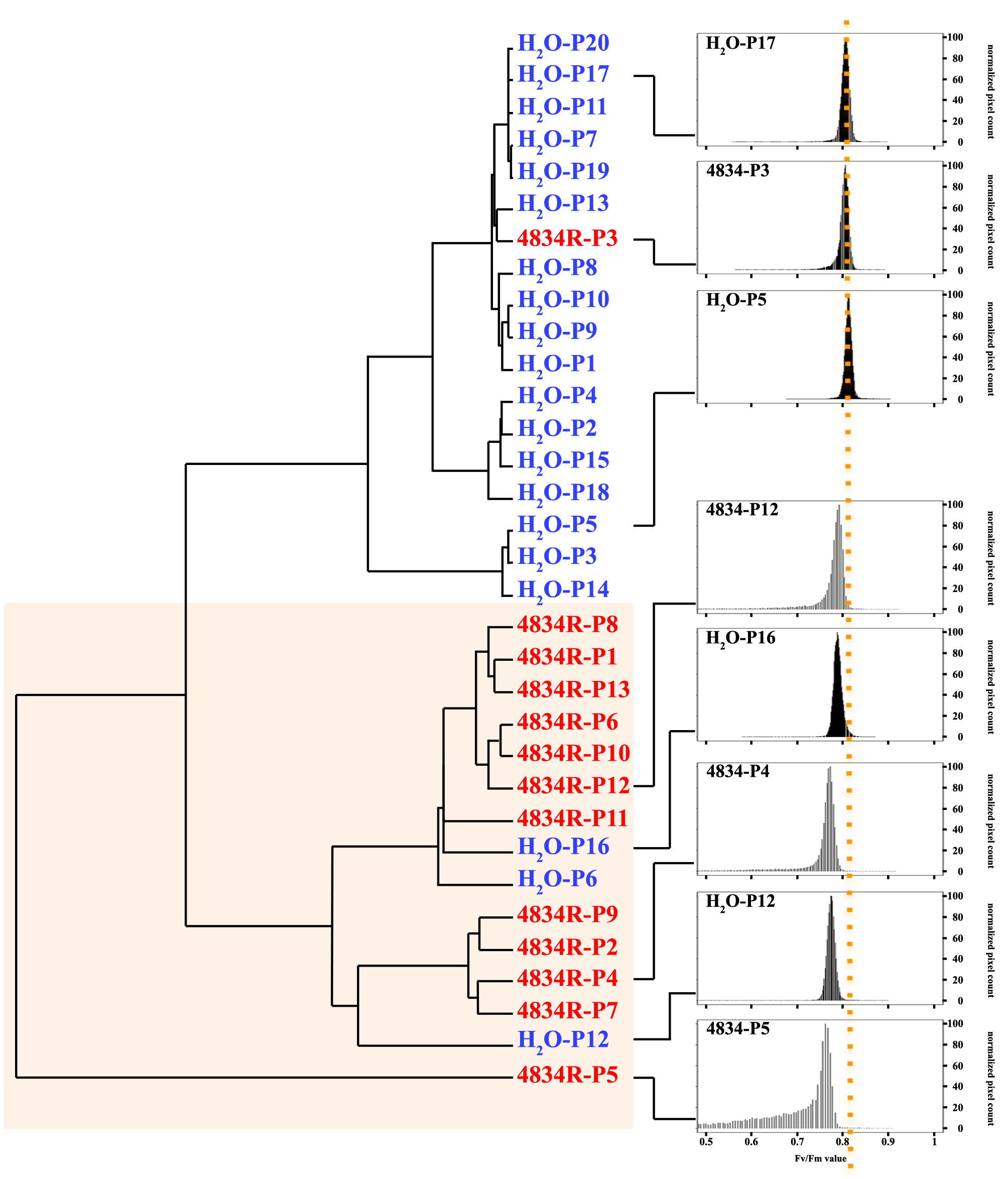

Supplement: Additional file 4: — Robustness of the clustering procedure. Bean leaflets of the cultivar Flavert inoculated with X. fuscans subsp. fuscans CFBP4834-R (1.106 CFU.ml−1) or mock were imaged for the fluorescence parameter Fv/Fm. A dendrogram (on the left) based on the histograms was built from the UPGMC agglomeration method. Globally, CFBP4834-R-inoculated leaflets (red) and mock-inoculated leaflets (blue) belong to different groups. Two main clusters can be observed. (i) Images of most of the mock-inoculated leaflet and of the CFBP4834-R-inoculated leaflet 4834R-p3 form a first cluster. The histograms (on the right) of these images are similar and present a peak around the Fv/Fm value of 0.8 (indicated by an orange dotted-line). (ii) Images of most of the CFBP4834R-inoculated leaflet and of the mock-inoculated leaflets H2O-p16, H2O-p6 and H2O-p12 form a second cluster. The histograms of these images are right-shifted with regard to those of the first cluster and can present different shapes. [file 13007_2015_68_MOESM4_ESM.jpeg]

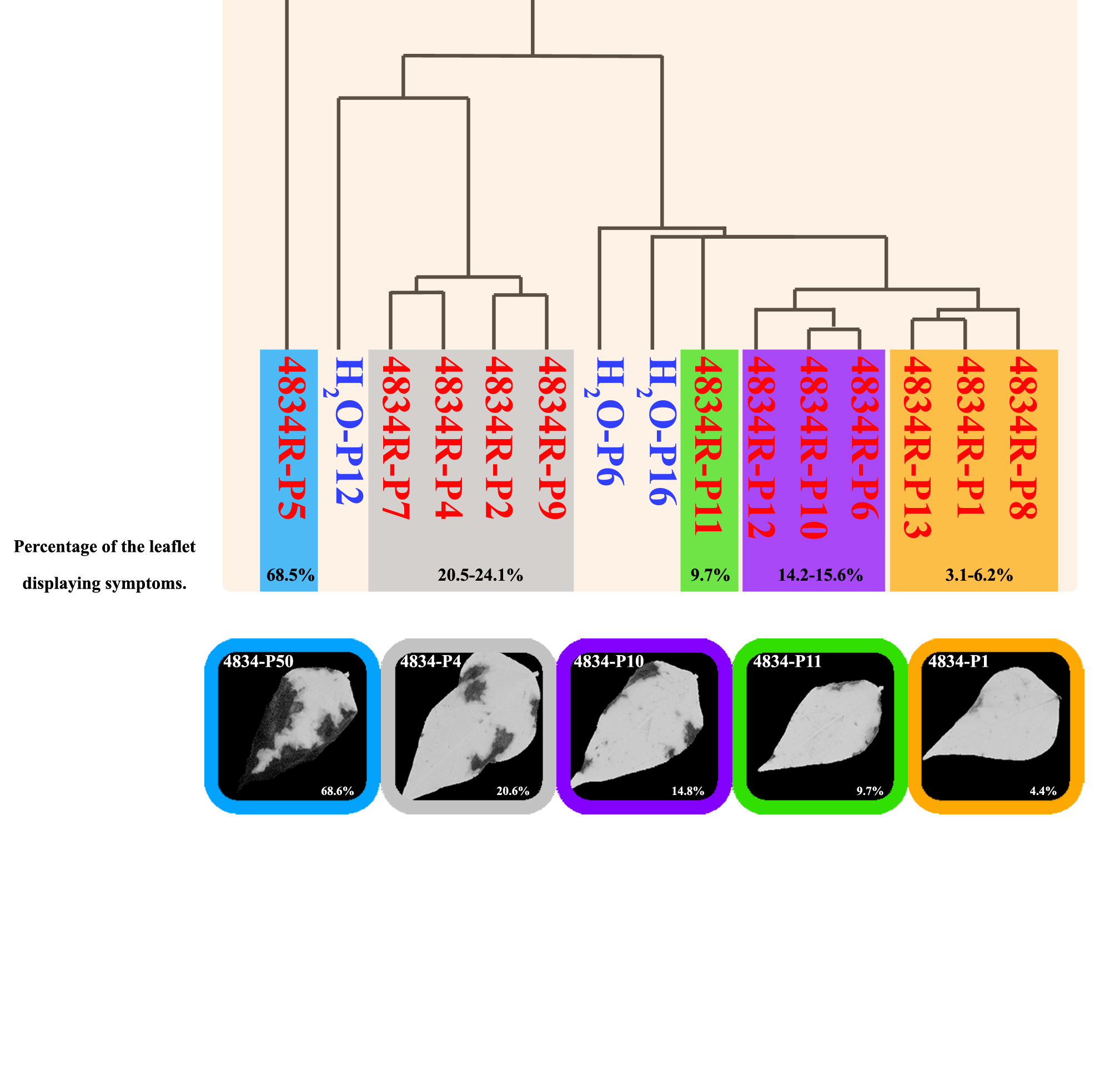

Supplement: Additional file 5: — Images of bean leaves cluster according to the severity of symptoms. Bean leaflets of the cultivar Flavert inoculated with X. fuscans subsp. fuscans CFBP4834-R (1.106 CFU.ml−1) or mock were imaged for the fluorescence parameter Fv/Fm. The figure represents an enlargement of the shaded part of the dendrogram found in Additional file 4. Five clusters of histograms can be defined according to the amount of symptoms (indicated by coloured boxes). The image of one leaflet representative of each group is displayed at the bottom. Low intensity pixels are displayed by high intensities of black and correspond to unhealthy tissues (the scale of intensities of Fv/Fm is displayed on the right of the images). The leaflet 4834R-p5 forms a full cluster and displays 68.5% of symptoms. The grey box is composed by the histograms of leaflets displaying between 20.5 and 24.1% of symptoms. The purple box is composed by the histograms of leaflets displaying between 14.2 and 15.6% of symptoms. The leaflet 4834R-p11 forms a full cluster and displays 9.7% of symptoms. The orange box is composed by the histograms of leaflets displaying less than 6.2% of symptoms. [file 13007_2015_68_MOESM5_ESM.jpeg]
